# Efficacy and safety of long-term treatment with lenalidomide and dexamethasone in patients with relapsed/refractory multiple myeloma

**DOI:** 10.1038/bcj.2014.77

**Published:** 2014-11-07

**Authors:** M A Dimopoulos, A S Swern, J S Li, M Hussein, L Weiss, Y Nagarwala, R Baz

**Affiliations:** 1 Department of Clinical Therapeutics, University of Athens School of Medicine, Athens, Greece; 2 Department of Biostatistics, Celgene Corporation, Summit, NJ, USA; 3 Department of Medical Affairs, Celgene Corporation, Summit, NJ, USA; 4 Department of Drug Safety, Celgene Corporation, Summit, NJ, USA; 5 Department of Hematologic Malignancies, H Lee Moffitt Cancer Center and Research Institute, Tampa, FL, USA

## Abstract

Data from two randomized pivotal, phase 3 trials evaluating the combination of lenalidomide and dexamethasone in relapsed/refractory multiple myeloma (RRMM) were pooled to characterize the subset of patients who achieved long-term benefit of therapy (progression-free survival ⩾3 years). Patients with long-term benefit of therapy (*n*=45) had a median duration of treatment of 48.1 months and a response rate of 100%. Humoral improvement (uninvolved immunoglobulin A) was more common in patients with long-term benefit of therapy (79% vs 55% *P*=0.002). Significant predictors of long-term benefit of therapy in multivariate analysis were age<65 years (*P*=0.03), β2-microglobulin <2.5 mg/l (*P*=0.002) and fewer prior therapies (*P*=0.002). The exposure-adjusted incidence rate (EAIR) of grade 3–4 neutropenia was lower in patients with long-term benefit of therapy (13.9 vs 38.2 per 100 patient-years). The EAIR for invasive second primary malignancy was the same in patients with long-term benefit of therapy and other patients (1.7 per 100 patient-years). These findings indicate that patients with RRMM can experience long-term benefit with lenalidomide and dexamethasone treatment with manageable side effects.

## Introduction

In two randomized phase 3 pivotal trials (MM-009 and MM-010), the oral IMiDs immunomodulatory drug lenalidomide was shown to improve outcomes when combined with dexamethasone in patients with relapsed/refractory multiple myeloma (RRMM), compared with dexamethasone alone.^1, 2^ In a pooled analysis of data from these trials, lenalidomide and dexamethasone significantly extended the median time to progression (TTP) (13.4 vs 4.6 months; *P*<0.001).^3^ Median overall survival (OS) was also improved (38.0 vs 31.6 months; *P*=0.045), despite the fact that 48% of patients assigned to dexamethasone alone had crossed over to receive lenalidomide-based therapy.^3^ Several factors have been associated with improved outcomes in patients treated with lenalidomide and dexamethasone, including early use of lenalidomide and dexamethasone immediately after the first-line therapy,^4^ reduction of lenalidomide dose after 12 months of full-dose therapy,^5^ increased depth of response^6^ and prolonged duration of treatment.^7^ Here we present the efficacy and safety of long-term treatment with lenalidomide and dexamethasone in patients from MM-009 and MM-010, and compare the characteristics of patients with long-term benefit of therapy (progression-free survival (PFS) ⩾3 years) with other patients (on study <3 years) to identify factors that may help predict treatment outcomes. Changes in immunoglobulin (Ig) levels were also assessed, as increased immunoglobulin levels have been observed in up to 20% of patients receiving long-term treatment with lenalidomide, and this increase in uninvolved immunoglobulin levels—a possible marker of improved immunity—has been associated with long-term clinical benefit.^8^

## Materials and methods

### Study design

Clinical study protocols of MM-009 and MM-010 were similar and have been described in detail elsewhere.^1, 2^ In brief, patients (⩾18 years of age) were randomized to oral lenalidomide (25 mg/day) or placebo, given on days 1–21 of each 28-day cycle, and all received oral dexamethasone (40 mg) on days 1–4, 9–12 and 17–20 for the first four cycles and on days 1–4 only thereafter. Eligible patients had measurable progressive disease after one or more myeloma treatments and serum creatinine <2.5 mg per 100 ml. They were excluded if they had disease that was resistant to total monthly doses of dexamethasone of >200 mg.

For patients who developed grade 3 or 4 adverse events, the dose of lenalidomide was withheld until the event resolved, and treatment was restarted using a dose of 15 mg/day, with further reductions in 5 mg decrements as needed. For those with isolated grade 3 or 4 neutropenia, subcutaneous granulocyte colony-stimulating factor (GCSF) (5 μg/kg/day) was given with the first dose-reduction step. Prophylactic anticoagulation was not warranted at the time of trial design per existing guidelines.

Results of a preplanned interim analysis by the data monitoring committee indicated that the O'Brien–Fleming boundary for superiority in TTP was crossed, favoring lenalidomide over placebo. At that time, the study was unblinded and patients assigned to placebo were allowed to receive lenalidomide or lenalidomide plus dexamethasone immediately or at the time of progression.

### Assessments

For this analysis, data on patients treated with lenalidomide and dexamethasone were pooled from MM-009 (*n*=177)^1^ and MM-010 (*n*=176).^2^

Patients with long-term benefit of therapy included all subjects with PFS ⩾3 years. Other patients included subjects on study <3 years (discontinuation due to progressive disease, adverse events, lost to follow-up, censored or death); PFS was defined as the time from randomization until the date of progression or death from any cause, whichever occurred first. The rationale to report on the 3-year data cut-off was because it represented the longest term data. Sensitivity analyses were performed at 1 year and 2 years and consistent results were seen at each data cut. TTP was defined as the time from randomization to the date of first assessment showing progression. OS was calculated as the time from randomization to death from any cause. Response was assessed every 4 weeks according to modified European Group for Blood and Marrow Transplantation criteria.^9^ Adverse events were graded according to the National Cancer Institute's Common Toxicity Criteria, version 2.0.^10^

Levels of uninvolved IgA, IgG and IgM were assessed at baseline and monthly during the monitoring period and compared with IgA levels in non-IgA type multiple myeloma (MM). Humoral improvement was defined as an increase from the baseline level of the uninvolved immunoglobulin to at least the lower-limit of normal, or a 25% increase in value from baseline. Comparisons were made between treatment responders and nonresponders according to their humoral response, which was defined as improvement achieved at ⩾3 post-baseline cycles. Uninvolved IgA in patients with non-IgA type MM was the only polyclonal immunoglobulin to show recovery during the monitoring period; therefore, subsequent analyses focused on IgA.

Average dosages of lenalidomide and dexamethasone were calculated as the total dose taken during the study period divided by the total number of days taking each drug. Dose compliance was defined as the total dose taken divided by the expected total dose based on the number of days.

### Statistical analyses

In this retrospective *post-hoc* pooled analysis, time-to-event analyses employed the Kaplan–Meier method and log-rank test for comparison between the groups. Summary statistics for baseline characteristics, treatment duration, dosing and adverse events are provided in order to better characterize the patients—both those with long-term benefit of therapy and other patients. Exposure adjusted incidence rates (EAIR) were calculated per 100 person-years=100 × *n*/*T*, where *n*=number of subjects with specified event and *T*=total person-years; person-years are calculated as the time from the first dose date to the onset date of first event (for subjects with event) and to the date of last dose (for subjects without an event). The 95% confidence interval for EAIR per 100 person-years is calculated using the method by RG Miller: EAIR × exp(±1.96/*n*^[1/2]^). Second primary malignancies (SPM) were defined using the Medical Dictionary for Regulatory Activities terms found under the System Organ Class Neoplasms.^11^

Several baseline variables and prognostic factors including age, time from diagnosis, number of prior therapies, prior stem cell transplantation, prior thalidomide therapy, β2-microglobulin levels, hemoglobin, platelet, albumin levels, lymphocytes, creatinine clearance and disease stage were evaluated in univariate logistic regression analyses to identify the prognostic factors associated with long-term benefit of therapy. Cytogenetic abnormalities were not routinely recorded and were therefore not included in the analysis. All factors with a 0.15 significance level in the univariate model were included in multiple logistic models. A best model was selected by fitting all possible models and using Akaike information criterion to select the best model and determine the covariates significant in long-term benefit from therapy.

## Results

### Demographics and clinical characteristics

Of the 353 patients treated with lenalidomide and dexamethasone, 45 (13%) had long-term benefit of therapy (Table 1). Compared with other patients, those with long-term benefit of therapy were younger (median age 58 years vs 64 years) and had lower β2-microglobulin levels (median 2.5 mg/l vs 3.6 mg/l). Time from diagnosis, myeloma stage and performance status were comparable between the two groups. The median number of lines of prior therapy was two (range 1–3) in both groups, although patients with long-term benefit of therapy were more likely to have undergone prior autologous stem cell transplantation (67% vs 57%), and less likely to have received thalidomide (27% vs 37%).

### Efficacy

The median follow-up was 49.7 months for patients with long-term benefit of therapy and 27.2 months for other patients. As expected, the median duration of treatment was longer in patients with long-term benefit of therapy (48.1 months (range 25.3–58.3 months)) compared with other patients (7.6 months (range 0.03–58.2 months)). Of the 45 patients who had long-term benefit of therapy, 44 (98%) were still on therapy after 3 years. All patients with long-term benefit of therapy achieved a partial response or better (100%), compared with 167 other patients (54%); the complete response rate was 56% and 11%, respectively (Table 2). Median time to first response was similar in both groups, but the median duration of response was higher in patients with long-term benefit of therapy (not reached (95% confidence interval 44.6–not reached) vs 9.9 months (95% confidence interval 7.9–12.5 months)).

**Figure 1:**
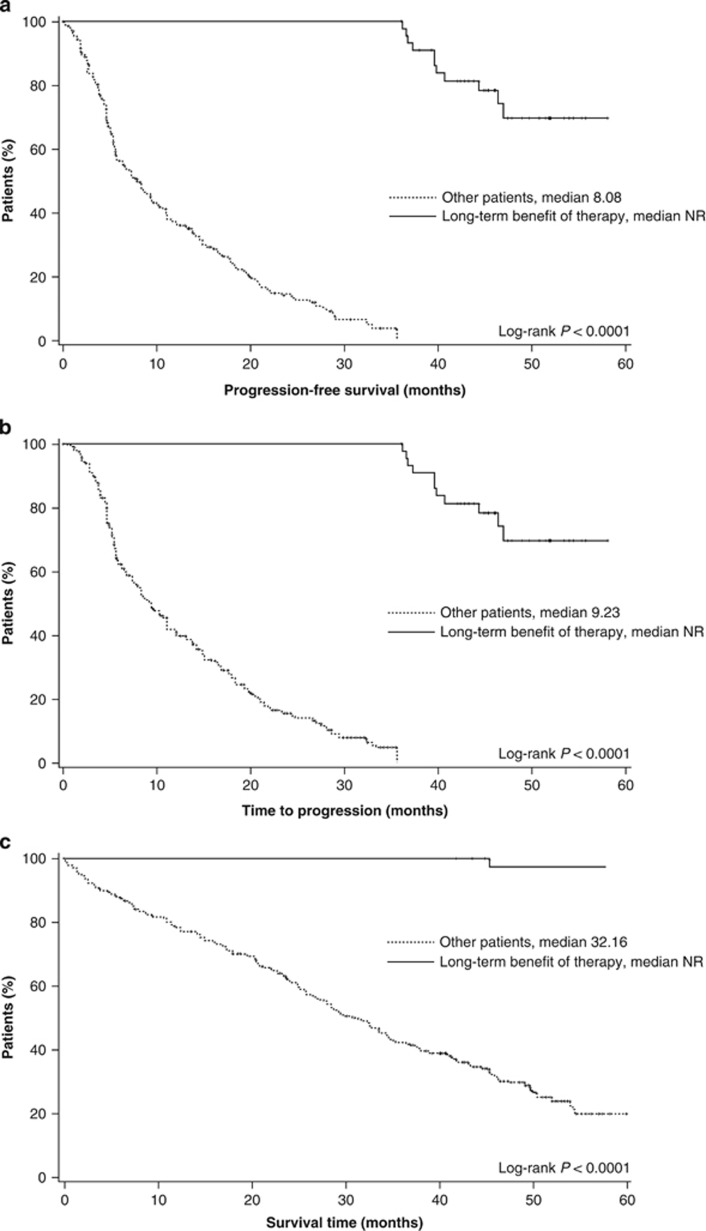
Kaplan–Meier estimates of PFS (**a**), TTP (**b**) and OS (**c**). NR, not reached.

Median PFS, TTP and OS were superior in patients with long-term benefit of therapy compared with other patients (Figure 1). With a median follow-up of 49.7 months for patients with long-term benefit of therapy, while the median PFS, TTP and OS had not been reached.

The results of the multivariate analysis showed that the odds of having long-term benefit of therapy were approximately doubled in younger patients (age ⩽65 years) compared with older patients, and for each decrease in the number of prior anti-myeloma therapies (Table 3). The odds of having long-term benefit of therapy were about tripled for patients with β2-microglobulin <2.5 mg/l. Whereas disease stage, hemoglobin levels, albumin levels and creatinine clearance showed significant prognostic association in univariate regression analysis, they were not significant in the multivariate analysis. Factors such as prior autologous stem cell transplantation, prior thalidomide therapy, time from myeloma diagnosis and baseline lymphocytes were not significant prognostic factors in either the univariate or the multivariate model.

### Humoral improvement

**Figure 2:**
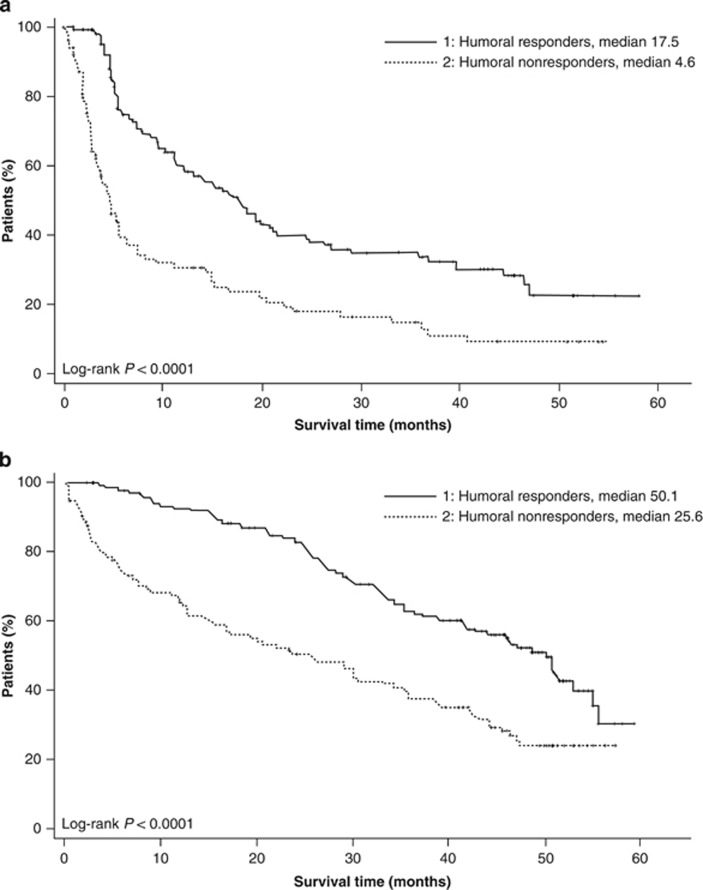
Kaplan–Meier estimates of PFS (**a**) and OS (**b**) according to humoral response in the overall population.

Of the 353 patients treated with lenalidomide and dexamethasone in the trials, 274 (79%) had non-IgA type MM; of those, 158 (58%) had an increase in uninvolved IgA (humoral response). Median PFS was significantly longer in humoral responders than in nonresponders (17.5 vs 4.6 months; *P*<0.0001) (Figure 2a). Similarly, median OS was significantly longer in humoral responders than in nonresponders (50.1 vs 25.6 months; *P*<0.0001) (Figure 2b).

**Figure 3:**
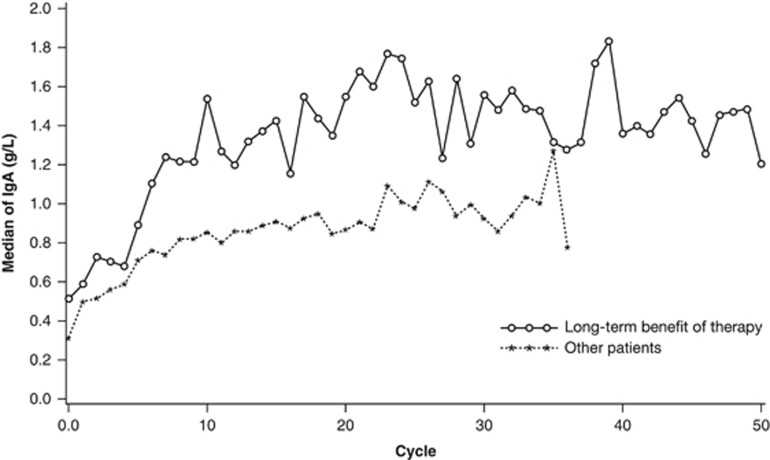
Median IgA (g/l) by progression-free survival group and cycle.

Figure 3 shows median levels of uninvolved IgA during the study. Overall, the median IgA increased during cycles 1–10 and then stabilized for the rest of the study period.

Humoral response rate was significantly higher in patients with long-term benefit of therapy (79%) than in other patients (54%) (*P*=0.006). However, median time to humoral response was the same in both groups (1 month). The magnitude of increase over time was greater in patients with long-term benefit of therapy (Figure 3).

### Safety and management

The incidence of grade 3 and 4 adverse events was generally comparable in patients with long-term benefit of therapy and other patients. Neutropenia and infection are very common adverse events in patients treated with lenalidomide as shown previously; the EAIR in this study was lower in patients with long-term benefit of therapy than in other patients (13.9 vs 38.2 per 100 patient-years for neutropenia and 10.0 vs 27.6 per 100 patient-years for infection) (Table 4). The EAIR of grade 3 or 4 non-hematological adverse events was generally low, and lower in patients with long-term benefit of therapy; they included deep-vein thrombosis/pulmonary embolism (1.7 vs 14.3 per 100 patient-years), fatigue (1.7 vs 7.8 per 100 patient-years), neuropathy (1.1 vs 4.8 per 100 patient-years), diarrhea (1.1 vs 3.1 per 100 patient-years) and constipation (0 vs 2.7 per 100 patient-years).

### Second primary malignancies

Invasive SPM were reported in eight patients treated with lenalidomide and dexamethasone and two patients treated with placebo and dexamethasone (Table 5). Hematologic SPM included two cases of myelodysplatic syndromes in the lenalidomide and dexamethasone group. The remaining six cases in the lenalidomide and dexamethasone group and two cases in the placebo and dexamethasone group were solid tumors. Of the eight cases of invasive SPM in patients treated with lenalidomide and dexamethasone, three occurred in patients with long-term benefit of therapy (6.7%) and five in other patients (1.6%). Among those treated with lenalidomide and dexamethasone, there was no difference in EAIR for invasive SPM between patients with long-term benefit of therapy and other patients (1.7 per 100 patient-years for both).

### Lenalidomide dose

The average dosage of lenalidomide was slightly lower 20.4 mg (range 5.9–25.0 mg) in patients with long-term benefit of therapy and 22.1 mg (range 7.0–25.3 mg) in the other patients. Lenalidomide dose compliance rates were 0.67 and 0.74, respectively. The average dosage of dexamethasone was 35.5 mg (range 20.7–40.0 mg) in patients with long-term benefit of therapy vs 38.3 mg (range 20.7–40.0 mg) in other patients; the dexamethasone dose compliance rates were 0.72 and 0.78, respectively. Lenalidomide dose reductions were relatively common in both groups, occurring in 24 patients with long-term benefit of therapy (53%) and 123 other patients (40%). The median time to lenalidomide dose reduction was markedly longer in patients with long-term benefit of therapy than for other patients (15.5 months (range 1.4–45.0 months) vs 3.3 months (range 1.0–29.7 months)). Sixty-two percent of lenalidomide dose reductions occurred after the first year of study treatment in patients with long-term benefit of therapy, whereas 84% of dose reductions occurred within the first year of study treatment in other patients. Of the 39 patients with long-term benefit of therapy who had no lenalidomide dose reductions during the first four treatment cycles, 12 (30.8%) had dexamethasone dose reductions. Of the 243 other patients who had no lenalidomide dose reductions during the first four treatment cycles, 61 (25.1%) had dexamethasone dose reductions.

The rate of treatment discontinuation was higher in the other patients population. Disease progression was the most common reason for discontinuation in other patients (176 of 308 patients; 57.1%), whereas only three patients with long-term benefit of therapy (6.7%) discontinued treatment—after 3 years and due to disease progression. Adverse events led to treatment discontinuation in 61 other patients (19.8%), compared with five patients with long-term benefit of therapy (11.1%).

## Discussion

This analysis showed that a proportion of patients with RRMM treated with lenalidomide and dexamethasone achieved an extended PFS interval of 3 years or more. These patients had a high response rate (100%), a markedly increased treatment duration (48.1 months) and median PFS, TTP and OS that were not reached with a median follow-up of ~4 years. Predictors of long-term benefit of therapy were: younger age (<65 years), fewer prior anti-myeloma therapies and β2-microglobulin <2.5 mg/l. The identification of known prognostic factors suggests that disease biology remains an important determinant of outcomes. Notably, whereas patient age and β2-microglobulin level at baseline cannot be modified, the number of prior anti-myeloma therapies can; several reports support the benefit of using lenalidomide and dexamethasone early in the course of the disease.^4, 12, 13, 14, 15^

The present findings are consistent with previous *post-hoc* analyses of MM-009 and MM-010 data that support continued treatment with lenalidomide and dexamethasone.^6, 7^ One analysis showed that the depth of response improved with continued treatment: 50% of patients who achieved a partial response as their initial response achieved a complete or very good partial response with continued treatment.^6^ Median OS was significantly higher in patients who achieved a complete or very good partial response than in those who had a partial response (not reached vs 44.2 months; *P*=0.021). In a second analysis of patients who achieve a partial response or better with lenalidomide and dexamethasone, there was a trend toward improved OS in patients who continued to receive therapy (median lenalidomide dose 20.5 mg), compared with patients who discontinued treatment for reasons other than disease progression (50.9 months vs 35.0 months; *P*=0.0594).^7^ Although MM-009 and MM-010 were not designed to specifically evaluate continued therapy, these findings support continuing treatment with lenalidomide and dexamethasone in responding patients.

This study is limited by the *post-hoc* nature of the analysis and the relatively small patient population with long-term benefit of therapy. Although it is known that cytogenetic risk profile has an impact on prognosis with lenalidomide and dexamethasone treatment, these data were not available.^16^ However, the present results are generally consistent with findings from smaller, single-centre retrospective studies.^17, 18^ In a report based on 50 patients who received long-term treatment with lenalidomide and dexamethasone (median treatment duration 3 years), Fouquet *et al*^17^ noted a significant improvement in TTP rate in patients with longer exposure to lenalidomide (37-month TTP rate 78% vs 91% for patients with lenalidomide exposure 2–3 years vs >3 years, respectively; *P*=0.025). Similarly, in a report based on 67 patients with RRMM, median OS was significantly higher in patients who received lenalidomide and dexamethasone for >1 year, compared with patients who stopped early for reasons other than disease progression (42.9 vs 20.5 months; *P*=0.0003).^18^

In this long-term analysis, patients with a humoral response experienced significantly longer PFS and OS compared with those without improvement in the uninvolved IgA (*P*<0.0001 for both). Previous studies have also reported an association between levels of IgA and survival outcomes.^8, 19^ A retrospective analysis of 104 patients who received lenalidomide for longer than 6 months showed that those with uninvolved IgA above the median level of 34 mg/dl (0.34 g/l) had prolonged PFS (*P*<0.01).^8^ The potential mechanisms for this correlation are still unclear. The benefits of long-term treatment with lenalidomide could be mediated by immunomodulation via polyclonal immune activation,^8^ or suppressing infections which are a manifestation of active myeloma.^20^

The safety profile of long-term treatment with lenalidomide and dexamethasone was acceptable, with no evidence of cumulative adverse events. The most common grade 3 or 4 adverse event was neutropenia, which occurred in over half of the patients with long-term benefit of therapy. The EAIR of grade 3–4 neutropenia was lower in patients with long-term benefit of therapy than in the other patients (13.9 vs 38.2 per 100 patient-years). It should also be noted that use of G-CSF was relatively limited in the MM-009 and MM-010 studies compared with current practice.^21, 22^ The EAIR of grade 3–4 venous thromboembolic events was lower in patients with long-term benefit of therapy than in other patients (1.7 vs 14.3 per 100 patient-years). Although prophylaxis with aspirin or anticoagulants was not mandated in the MM-009 and MM-010 trials, it has since been shown to effectively reduce the risk of venous thromboembolism event in patients treated with lenalidomide-based therapy.^15, 23, 24^ No difference in the EAIR of invasive SPM was observed between patients with long-term benefit of therapy and other patients (1.7 per 100 patient-years). This is consistent with other findings^17^ and comparable to expected background rates in an elderly population: the Surveillance, Epidemiology and End Results Program reports age-adjusted incidence rates for invasive cancers of 0.6 among persons 50–54 years of age, 0.8 among persons 55–60 years of age, 1.2 among persons 60–64 years of age and 2.1 among persons 65 years of age or older.^25^ This further supports the positive benefit/risk profile of lenalidomide in RRMM.^11^

In summary, patients with RRMM can experience long-term benefit of therapy with lenalidomide and dexamethasone. Results of this analysis support continuing treatment in responding patients with appropriate management of neutropenia and other adverse events.

## Figures and Tables

**Table 1:** Patient baseline characteristics

*Characteristic*^a^	*Patients with long-term benefit of therapy (*N*=45)*	*Other patients (*N*=308)*
Age, years (range)	58 (33–75)	64 (35–86)
⩾65, *n* (%)	11 (24.4)	150 (48.7)
<65, *n* (%)	34 (75.6)	158 (51.3)
Male, *n* (%)	28 (62.2)	182 (59.1)
Time from diagnosis, years (range)	3.1 (0.4–13.1)	3.3 (0.5–15.7)
		
*ISS*, n *(%)*		
* *I or II	37 (88.1)	229 (76.1)
* *III	5 (11.9)	72 (23.9)
		
*ECOG performance status*, n *(%)*		
* *0	19 (42.2)	133 (43.2)
* ⩾*1	26 (57.8)	175 (56.8)
		
*β2-microglobulin, mg/l*		
* *⩾2.5, *n*/*N* (%)	21/42 (50.0)	239/302 (79.1)
* *<2.5, *n*/*N* (%)	21/42 (50.0)	63/302 (20.9)
		
Creatinine clearance, ml/min	85.7 (21.2–161.6)	75.3 (17.3–209.8)
		
*Number of lines of prior therapy,* n *(%)*		
* *0–1	22 (48.9)	111 (36.0)
* *2	20 (44.4)	118 (38.3)
* *⩾3	3 (6.7)	79 (25.6)
* *Prior thalidomide, *n* (%)	12 (26.7)	115 (37.3)
* *Prior ASCT, *n* (%)	30 (66.7)	177 (57.5)
* *IgA type MM, *n* (%)	8 (17.8)	67 (21.8)

Abbreviations: ASCT, autologous stem cell transplantation; ECOG, Eastern Cooperative Oncology Group; Ig, immunoglobulin; ISS, International Staging System; MM, multiple myeloma.

a All values are median (range) unless otherwise stated.

**Table 2:** Response rate, time to response and duration of response

*Response*	*Patients with long-term benefit of therapy (*N*=45)*	*Other patients (*N*=308)*	*All patients (*N*=353)*
Overall response (PR or better), *n* (%)	45 (100.0)	167 (54.2)	212 (60)
CR	25 (55.6)	33 (10.7)	58 (16.4)
nCR/VGPR	8 (17.8)	17 (5.5)	25 (7.1)
PR	12 (26.7)	117 (38.0)	129 (36.5)
SD, *n* (%)	0 (0.0)	100 (32.5)	100 (28.3)
PD, *n* (%)	0 (0.0)	11 (3.6)	11 (3.1)
Not evaluable, *n* (%)	0 (0.0)	30 (9.7)	30 (8.5)
Median time to first response, months (range)^a^	2.8 (1.9, 16.6)	2.7 (1.4, 18.2)	2.8 (1.4, 18.2)
Median duration of response, months (95% CI)^b^	NR (44.6, NR)	9.9 (7.9, 12.5)	15.5 (12.0, 19.7)

Abbreviations: CI, confidence interval; CR, complete response; nCR, near complete response; NR, not reached; PD, progressive disease; PR, partial response; SD, stable disease; VGPR, very good partial response.

a For responding patients only.

b Kaplan–Meier estimates of median (95% CI).

**Table 3:** Logistic regression analysis of predictors of patients with long-term benefit of therapy

P*<0.15*	*Univariate*		*Multivariate*	
	*Odds ratio*^a^ *(95% CI)*	P-*value*	*Odds ratio (95% CI)*	P*-value*
ISS group ((I & II) vs III)	2.33 (0.88, 6.14)	0.088		
Hemoglobin (for each unit increase)	1.17 (0.98, 1.40)	0.085		
β2-microglobulin level	0.68 (0.54, 0.86)	0.001		
β2-microglobulin group (<2.5 vs ⩾2.5 mg/l)	3.79 (1.95, 7.38)	<0.001	3.02 (1.51, 6.03)	0.002
Age (for each year increase)	0.95 (0.92, 0.98)	0.001		
Age group (<65 vs ⩾65 years)	2.77 (1.32, 5.79)	0.007	2.36 (1.09, 5.12)	0.03
Albumin (for each unit increase)	1.99 (1.11, 3.57)	0.021		
Creatinine clearance	1.01 (1.00, 1.02)	0.006		
Number of previous anti-myeloma therapies^b^	0.50 (0.34, 0.74)	<0.001	0.52 (0.35, 0.78)	0.002

Abbreviations: CI, confidence interval; ISS, International Staging System.

a The odds of being in patients with long-term benefit of therapy vs other patients.

b For every increase in the number of prior anti-myeloma therapies, the odds are reduced by half.

**Table 4:** Grade 3–4 adverse events

*Event*	*Patients with long-term benefit of therapy (*N*=45)*	*Other patients (*N*=308)*	*All patients (*N*=353)*
*Hematologic*			
Neutropenia, *n* (%)	25 (55.6)	112 (36.4)	137 (38.8)
EAIR (95% CI)	13.9 (9.4, 20.6)	38.2 (31.7, 45.9)	29 (24.5, 34.3)
Anemia, *n* (%)	7 (15.6)	38 (12.3)	45 (12.7)
EAIR (95% CI)	3.9 (1.9, 8.2)	13.0 (9.4, 17.8)	9.5 (7.1, 12.7)
Thrombocytopenia, *n* (%)	6 (13.3)	42 (13.6)	48 (13.6)
EAIR (95% CI)	3.3 (1.5, 7.4)	14.3 (10.6, 19.4)	10.2 (7.7, 13.5)
Febrile neutropenia, *n* (%)	2 (4.4)	9 (2.9)	11 (3.1)
EAIR (95% CI)	1.1 (0.3, 4.5)	3.1 (1.6, 5.9)	2.3 (1.3, 4.2)
			
*Non-hematologic*			
Infection, *n* (%)	18 (40.0)	81 (26.3)	99 (28.0)
EAIR (95% CI)	10.0 (6.3, 15.9)	27.6 (22.2, 34.3)	20.9 (17.2, 25.5)
DVT/PE, *n* (%)	3 (6.7)	42 (13.6)	45 (12.7)
EAIR (95% CI)	1.7 (0.5, 5.2)	14.3 (10.6, 19.4)	9.5 (7.1, 12.7)
Fatigue, *n* (%)	3 (6.7)	23 (7.5)	26 (7.4)
EAIR (95% CI)	1.7 (0.5, 5.2)	7.8 (5.2, 11.8)	5.5 (3.7, 8.1)
Neuropathy^a^, *n* (%)	2 (4.4)	14 (4.5)	16 (4.5)
EAIR (95% CI)	1.1 (0.3, 4.5)	4.8 (2.8, 8.1)	3.4 (2.1, 5.5)
Diarrhea, *n* (%)	2 (4.4)	9 (2.9)	11 (3.1)
EAIR (95% CI)	1.1 (0.3, 4.5)	3.1 (1.6, 5.9)	2.3 (1.3, 4.2)
Constipation, *n* (%)	0 (0.0)	8 (2.6)	8 (2.3)
EAIR (95% CI)	0 (0, 0)	2.7 (1.4, 5.5)	1.7 (0.8, 3.4)

Abbreviations: CI, confidence interval; DVT, deep-vein thrombosis; EAIR, exposure-adjusted incidence rate per 100 patient-years; PE, pulmonary embolism.

a Neuropathy includes neuropathy, peripheral neuropathy, peripheral sensory neuropathy and polyneuropathy.

**Table 5:** Incidence of second primary malignancy

*SPM,* n *(%)*	*Patients with long-term benefit of therapy (*N*=45)*	*Other patients (*N*=308)*	*All lenalidomide/dexamethasone patients (*N*=353)*
Total invasive^a^, *n* (%)	3 (6.7)	5 (1.6)	8 (2.3)
EAIR (95% CI)	1.7 ( 0.6, 5.3)	1.7 ( 0.7, 4.1)	1.7 (0.9, 3.4)
MDS, *n* (%)	0	2 (0.6)	2 (0.6)
EAIR (95% CI)	0 (0, 0)	0.7 (0.2, 2.7)	0.4 (0.1, 1.7)
Solid tumor, *n* (%)	3 (6.7)	3 (1.0)	6 (1.7)
EAIR (95% CI)	1.7 (0.6, 5.3 )	1.0 (0.3, 3.2)	1.3 (0.6, 2.9)
Non-melanoma skin cancer, *n* (%)	2 (4.4)	9 (2.9)	11 (3.1)
EAIR (95% CI)	1.1 (0.3, 4.6)	3.2 (1.6, 6.1)	2.4 (1.3, 4.3)

Abbreviations: CI, confidence interval; EAIR, exposure-adjusted incidence rate per 100 patient-years; MDS, myelodysplastic syndrome; SPM, second primary malignancy.

a Includes MDS and solid tumors.

## Notes

MAD has had a consultant role for, and has received honoraria from, Celgene. MH, ASS, YN, JSL are Celgene employees and have equity ownership. RB has received research funding from Celgene.
